# Nasal mucus proteome and its involvement in allergic rhinitis

**DOI:** 10.1080/14789450.2020.1748502

**Published:** 2020-04-08

**Authors:** Peter Valentin Tomazic, Barbara Darnhofer, Ruth Birner-Gruenberger

**Affiliations:** a ENT-University Hospital, Medical University of Graz, Graz, Austria; b Diagnostic and Research Institute of Pathology, Diagnostic and Research Center of Molecular Medicine, Medical University of Graz, Graz, Austria; c BioTechMed-Graz, The Omics Center Graz, Graz, Austria; d Institute of Chemical Technologies and Analytics, Vienna University of Technology, Vienna, Austria

**Keywords:** Nasal mucus, proteomics, mass spectrometry

## Abstract

**Introduction**: Nasal mucus is the first line defense barrier against various pathogens including allergens. Proteins in nasal mucus maybe used as biomarkers for diagnosis or future therapeutic strategies. Proteomics opens the possibility to investigate whole human proteomes.

**Areas Covered**: We aimed to analyze the existing literature on nasal mucus and nasal secretions proteomic approaches especially in allergic rhinitis. A PubMed/Medline search was conducted entering the following keywords and combinations: “nasal mucus”, “nasal lavage fluid,” nasal secretions,” “nasal swabs,” “allergic rhinitis,” ”proteins,” and “proteomics.”

**Expert opinion**: The majority of studies focus on single proteins or protein groups mainly using ELISA techniques. Four studies met the criteria using mass spectrometry in the analysis of nasal mucus proteomes in rhinologic diseases. In these studies, 7, 35, 267, and 430 proteins were identified, respectively. These four studies are discussed in this review and put in relation to seven other proteomic studies that focus on nasal lavage fluid and nasal secretions obtained by swabs or filter paper. To put it in a nutshell, proteomics facilitates the investigation of the nasal secretome and its role in healthy and diseased state and as potential biomarkers for new diagnostic or therapeutic approaches.

## 1. Introduction

Nasal mucus is the first line defense barrier against a variety of inhaled pathogens. With around 12.000 L of air inhaled daily, the airways are confronted with 25 million particles an hour that need to be filtered and/or transported away [1–3]. The reaction of the epithelium to this challenge is the production of nasal mucus (and also mucus in the lower airways), which is a viscoelastic gel covering the epithelial surface as a film. It functions as a physical barrier against particles, irritants, microbes, viruses, but also food and liquids. Pathogens are trapped in the mucus and transported away through mucociliary clearance of the beating ciliated epithelium underneath the mucus layer. Mucus protects the epithelium from dehydration, warms and humidifies inhaled air, neutralizes toxic gases and buffers the pH value in the airways. It also contains anti-microbial agents as well as immunoglobulins (Ig) exerting innate and adaptive immune responses.

### 1.1. Nasal mucus proteome

Nasal mucus consists of a variety of lipids, glycoconjugates, cells, cellular debris, and proteins. Nasal mucus proteins work as enzymes or enzyme inhibitors, antioxidants, antibacterial agents, and mediators. Well described and abundant proteins in nasal mucus are mainly antimicrobial proteins like lysozyme and lactotransferrin as well as secretory immunoglobulins IgA, IgE, IgG, and albumin. Other proteins include kallikrein, antiproteases, ß-glucuronidase, and α-galactosidase as described in early, mainly ELISA-based, studies [4,5]. Mucus proteins stem from epithelial cells, goblet cells of sero-mucous glands in the submucosal tissue, either by secretion or cell death. Cellular debris in the mucus can be detected by its DNA or nucleic products such as histones [3]. Another important contributor to the protein composition of the mucus is proteins exudated from plasma, which is regarded as a key defense mechanism [3,4,6,7]. Especially in pathological conditions like allergic rhinitis, lysozyme, and lactotransferrin were shown to be up-regulated after provocation with allergen [4]. These early studies already underlined the importance of the nasal mucus proteome in disease, but were restricted to selected distinct proteins or small groups of proteins.

The mucus itself comprises two phases, a more viscous gel-layer on the top and a less viscous sol-layer below, which is in close contact with the cilia of the epithelium. Through metachronous and synchronous movement of the cilia, the mucus is moved in a way that the cilia beat within the sol-phase conducting the momentum to the gel-phase. Under pathological conditions, when the mucus is thickened at the expense of the sol-phase, the ciliary beat is stopped and the mucociliary clearance is hampered [3,8]. A balance between the two phases is mandatory in a healthy condition so that trapped pathogens are transported into the upper digestive tract to be swallowed and degraded in the stomach or coughed or sneezed out.

Despite its important role in defending the nose and the entire organism against various pathogens, nasal mucus has been granted less attention than the nasal epithelium. Particularly, nasal mucus proteins, major constituents and functional units of this body fluid, have not been studied extensively. Potential therapeutic targets like surfactant protein (SP-A) or clara cell protein (CC10) have been addressed in mouse models or *in vitro* models by analyzing their expression in the epithelium [9,10]. SP-A is secreted by type II alveolar epithelial cells into pulmonary surfactant, where it is involved in host defense and immune regulation by inhibiting Th2 cell differentiation, reducing Th2 cytokine levels, and increasing Th1 cytokines. It was also identified in nasal mucosa by immunostaining and PCR, and exogenous application resulted in reduced IL-4 and IL-5 levels in ovalbumin-sensitized mice [9]. These protective effects of SP-A may have therapeutic potential in allergic rhinitis. CC10 is an immunosuppressive protein secreted by nasal epithelial cells upon allergen stimulation. Fexofenadine hydrochloride, an H1 histamine receptor blocker, increased CC10 levels in vitro suggesting that CC10 could be used as a predictor for the efficacy of the agent in the individual patient [10].

#### 1.1.1. Mucins

Proper viscoelasticity and fluidic properties of the mucus are mainly accounted to glycoproteins called mucins. Mucins have been implicated in many airway diseases and were predominantly studied in the epithelium, despite the fact that they exert their functions and also harmful potential in the mucus of the upper and lower airways [11]. Mucins comprise up to 2% of the net weight of nasal mucus. Mucins often contain multiple protein domains with extensive O-glycan attachment [3,12]. They are produced in goblet cells and submucosal glands and encoded by specific MUC genes. Twenty human MUC genes have been identified. However, in the respiratory tract, only nine of these are expressed, namely MUC1, MUC2, MUC4, MUC5AC, MUC5B, MUC7, MUC8, MUC11, and MUC13 [1,3]. In healthy individuals, MUC5B is mainly expressed in submucosal glands whereas MUC5AC is exclusively present in goblet cells. Lin et al. [13] showed that 2-aminoethoxydiphenyl borate (2-APB), a chemical that potentially inhibits intracellular calcium release through modification of TRP channels, reduced MUC5B secretion from submucosal glands whereas IL-33 enhanced its secretion into the nasal mucus of allergic rhinitis mouse models. 2-APB also reduced IL-4, IL-5, and IL-13 in nasal mucus as well as in the epithelium. However, the production of IL-33 was not influenced. Moreover, 2-APB compromised tight junctions thus negatively influencing epithelial barrier function.

Mucin secretion from goblet cells is differentiated by two mechanisms: a basal secretion and an upregulated secretion stimulated by extracellular triggers [3]. Inflammatory stimuli in allergic disease via Th2 cytokines, like IL-4, IL-9, and IL-13, trigger mucin production. IL-13 together with STAT6 furthermore causes mucous metaplasia in airway epithelium, which is also important in allergic rhinitis since a higher viscosity of nasal mucus leads to an impairment of mucociliary clearance. The trapped mucus enhances symptoms like nasal blockage and harbors the potential of superinfections and subsequent chronic rhinosinusitis [1]. Mucins are stored in intracellular granules after synthesis at the endoplasmatic reticulum and glycosylation at the Golgi apparatus. For exocytosis, the granules are moved to the apical cell surface, which is dependent on myristoylated alanine-rich C-kinase substrate (MARCKS). After phosphorylation of MARCKS, actin/myosin contracts and the granules fuse with the plasma membrane [3]. The importance of goblet cells and mucin production is evident in pathological conditions like COPD (chronic obstructive pulmonary disease), asthma, and CF (cystic fibrosis), where goblet cell hyperplasia is predominant. Goblet cell hyperplasia results from enhanced differentiation of basal progenitor cells into goblet cells. Apart from mucus overproduction as part of the disease pathology, goblet cell hyperplasia leads to a reduction of clara cells, which are the main precursors of goblet cell differentiation. Since clara cells produce important immunomodulatory, anti-inflammatory and anti-bacterial mediators, their reduction further aggravates disease progress and clinical course [3].

#### 1.1.2. Immunoglobulins

Many studies have focused on immunoglobulins in nasal secretions. Especially, the local production of IgE and its relevance in nasal mucus was investigated in detail. In pollen season and/or after provocation IgE antibodies in nasal secretions were found to be significantly higher in allergic rhinitis patients than in healthy controls [14–21]. However, a clear diagnostic or therapeutic benefit from these findings could not be deducted. Since plasma levels did not correlate with the levels measured in nasal mucus, the presence of IgE in nasal mucus could not be explained by simple plasma exudation. Other important immunoglobulins such as secretory IgA (s-IgA) are secreted into body fluids in the upper respiratory, urogenital, and gastrointestinal tract. They are also present in nasal mucus and bind to antigens entering the mucus thus reducing their absorption. Ig-deficient subjects show a greater absorption which becomes apparent in food allergy. S-IgA can exert anti-inflammatory properties and encase microorganisms, which then cannot bind to the epithelium, a mechanism, which can be augmented by mucins. Through clumping of bacteria which is antibody- and glycan-mediated their mucosal clearance is enhanced. Furthermore, s-IgA can enhance the activity of protective proteins like lactoferrin. IgG, on the other hand, may activate the complement system promoting inflammation and subsequent epithelial damage and disintegration [22].

#### 1.1.3. Kallikrein and kinins

Another focus was laid on the investigation of kallikrein and kinins in nasal mucus as mediators of the allergic reaction. After provocation, these molecules were significantly elevated along with histamine in nasal secretions. These studies were performed in the course of the up-coming 1st generation antihistamines and proved that topical application of the latter could ameliorate allergic symptoms and decrease these mediators [23–25]. Baraniuk later suggested that the muscarinic receptor antagonist ipratropium bromide could reduce glandular secretion into nasal mucus via the bradykinin system [26]. However, histamine release and activation of the kallikrein-kinin system is a primary consequence of IgE cross-linking and mast cell activation but does not necessarily reflect which processes occur in mucus during allergen challenge.

#### 1.1.4. Eosinophilic proteins

As of today, many studies have focused on symptomatic therapy and its influence on nasal secretions but, because of therapeutic failures, targeted therapies toward mucus proteins may be favorable [27]. The association of eosinophils to allergic rhinitis gave rise to the investigation of its major protein component eosinophilic cationic protein (ECP) in nasal mucus. Since eosinophilic infiltration of the mucosa is a sign of inflammation, secretion of ECP into the mucus could reflect disease severity and regulate the course of disease. Indeed, ECP was significantly elevated in allergic mucus [28–32]. As potent toxin, this protein is also involved in epithelial disruption acting from outside-in, i.e. from the mucus toward the mucosa. An association between elevated ECP and IL-5 was found by Kramer et al. [33] employing ELISA. Anti-IL-5 is thus a promising therapeutic agent, but large randomized placebo-controlled trials for allergic rhinitis are lacking as of today [34,35]. Similar findings are true for major basic protein (MBP), another highly abundant eosinophilic protein [36].

#### 1.1.5. Substance P and vasoactive intestinal polypeptide

Tonnesen et al. [37] studied substance P and vasoactive intestinal polypeptide (VIP) in nasal mucus. As these inflammatory mediators were found to be elevated in nasal secretions of allergic rhinitis, the authors suggested that antagonists could positively influence the disease. Raphael et al. [38] and Ichikawa et al. [39] investigated the source of proteins in allergic nasal mucus. Depending on allergen challenge proteins were secreted from local mucosal glands or by means of plasma exudation. When studying the nasal mucus proteome it is therefore important to consider plasma exudation as a potential source of proteins which may bias measured protein abundances and to exclude potential triggers of plasma exudation like capsaicin [40].

#### 1.1.6. Antimicrobial proteins

Despite their possible dual origin, albumin, lactoferrin, lysozyme, tryptase, substance P, Igs, complement, and cytokines next to the eicosanoids leukotrienes and prostaglandins could act as potential mucus markers of allergic disease and/or disease modifiers [5,38,39,41–47]. Albumin is a plasma protein and its main functions are the maintenance of the colloid osmotic pressure in the vessels and transport of hormones and fatty acids. Its presence in nasal mucus is due to increased plasma exudation and thus its increase is regarded as a response to inflammatory stimuli. Whether albumin itself has immune defense properties is unclear as of today [7,48]. Local glandular production of albumin in the nose is possible, however, these amounts appear to be neglectable and no clear evidence has been presented to support it.

Lactoferrin or lactotransferrin is an abundant protein in nasal mucus and mainly produced in nasal submucosal glands where it is stored and released on demand. The 78 kD protein exerts bacteriostatic and bactericidal functions first described in 1966 in bovine milk. The antimicrobial effect is mainly achieved through its iron-binding capacities. Iron is essential for bacterial growth and is taken up by microorganisms. Furthermore, it blocks the formation of complement factor C3 convertase and regulates granulocyte production influencing granulocyte colony-stimulating factor (GCSF). Its iron-binding properties are comparable to that of transferrin in serum. Not only in nasal secretions but also in other body fluids lactoferrin is present in high abundance: tears, bronchial mucus, urogenital secretions, pancreas, liver, and gastrointestinal tract, as well as in granules of neutrophils. The concentration of lactoferrin in nasal secretions is around 1 µg/ml, which can be dramatically increased upon provocation with methacholine or histamine. The local production and secretion of this protein upon stimulation make it an important defense protein in the nasal mucus [4].

Lysozyme was first described by Flemming in 1922 when he discovered the bactericidal capacities of nasal secretion in patients with rhinitis after incubation with bacterial cultures. It has a molecular weight of around 14 kD. Lysozyme is able to enzymatically degrade bacterial cell walls. It is found in kidneys, stomach glands, small intestine, paneth cells, lacrimal, and parotid glands, as well as other salivary glands, neutrophils and macrophages. In the lung lysozyme accounts for 5–6% of total protein. As well as lactoferrin, it is produced in the local nasal mucosal glands and its secretion can be enhanced by external stimuli [4].

Another antimicrobial protein is psoriasin investigated by Bryborn et al. [49] and Kvarnhammar et al. [50] by means of 2-dimensional gel electrophoresis and mass spectrometry and ELISA, respectively. Bryborn et al. postulated that this chemoattractant protein could be a potential biomarker in allergic mucus, whereas Kvarnhammar suggested a compromised antimicrobial defense since the protein was diminished in an allergic milieu and could not exert its immunomodulatory properties.

Three other antimicrobial protein families have been investigated in asthma, namely bactericidal/permeability-increasing protein (BPI), the palate, lung and nasal epithelium clone PLUNC family and defensins, yet their potential influence in allergic rhinitis remains to be determined. BPI is present in neutrophils, eosinophils, and also epithelial cells of the oral cavity. It is active against gram-negative bacteria and neutralizes lipopolysaccharide (LPS). As implied by its name, BPI increases the permeability of bacterial cell membranes. Because of its structural similarities, the PLUNC protein belongs to the BPI protein family. In humans, it is expressed by major salivary glands and mucosal glands in the upper respiratory tract including the nose. The PLUNC proteins are indirect antimicrobial agents through binding to bacteria and LPS, but the exact mechanism is still unknown [51–54].

Azurocidin is a bactericidal and antiviral serine protease with controversial reports on its proteolytic activity [55]. Its bactericidal activity is specific for gram-negative bacteria by also binding to LPS. Its antiviral potential stems from a disruption of the envelope or the capsid and is not dependent on disulfide bonds. Furthermore, it is released early from secretory granules of neutrophils and has regulatory effects on recruitment and activation of monocytes [51].

Defensins are antimicrobial peptides with a triple-ß-hairpin structure, six conserved disulfide-linked cysteine residues and are positively charged. They can be classified into two main families in humans, six alpha and four beta defensins [51,56]. They have antimicrobial, antiviral and antifungal properties, modify cell migration and maturation and trigger histamine and prostaglandin D_2_ release from mast cells [51].

#### 1.1.7. Protease inhibitors

Pollen proteases can degrade tight junctions [57] and subsequently harm the nasal epithelium. One hypothesis is that an imbalance of nasal mucus antiproteases could favor protease activity and influence the disease. Hamaguchi et al. [23] found that protease activity in acute sinusitis was high, potentially hampering the healing process, whereas in allergic rhinitis protease activity in mucus was weak. They only focused on functional activity of cathepsin B and L, both cysteine proteases, while distinct antiproteases and their potential functional defects were not addressed. Belkowski et al. [58] investigated secretory leukocyte protease inhibitor (SLPI), which is cleaved by chymase. SLPIs, as well as elafin, are potent serine protease inhibitors. SLPI inhibits cathepsin G and elastase whereas elafin inhibits elastase and proteinase 3 but not cathepsin G. Cathepsin G is a serine protease with bactericidal function through hydrolytic degradation of bacteria and extracellular matrix components. This mechanism cannot be completely hampered by protease inhibitors [51,59]. SLPI and elafin were found in epithelium and mucus of the upper respiratory tract. They have antibacterial and antifungal properties and are up-regulated during inflammatory processes. The ratio between the native and the cleaved form of SLPI reflects inflammatory activity, but its involvement in the pathophysiology of the disease has not been elucidated.

## 2. Proteomics of the nasal mucus

The clear advantage of untargeted proteomic techniques using mass spectrometry is the unbiased identification of the entire population of proteins in a body compartment (*i.e*. the proteome) enabling allocation of their origin and function without defining the targets beforehand as in ELISA, Western blotting, or other antibody-based targeted techniques (Table 1). As a part of the first-line defense barrier against harmful agents, nasal mucus proteins are most likely involved in physiological and pathological processes. Moreover, the nasal mucus proteome can serve as a reservoir for biomarkers facilitating differential diagnosis for allergic rhinitis, like vasomotor rhinitis, or as diagnostic adjuncts in allergic diagnostics [60–62]. Iguchi et al. found a 26kD protein as a marker to differentiate between allergic rhinitis and vasomotor rhinitis using SDS-PAGE; however, the protein remained to be identified [60]. Tewfik [63] followed by Badaai [64] used a quantitative proteomic approach based on isobaric labeling and multiple reaction monitoring (MRM) to investigate the nasal mucus proteome in chronic rhinosinusitis (CRS) vs a control group of healthy volunteers. Patients were diagnosed according to guidelines, endoscopy, and paranasal sinus CT scans. If they received prior surgery and/or medication they were excluded. Under endoscopic visualization, nasal mucus was collected using a suction trap. No protease inhibitors were used to prevent interference with the trypsin digestions required for MS-based peptide sequence identification. Samples. Digested samples were tagged with iTRAQ and separated by two-dimensional capillary liquid chromatography (capLC). For mass spectrometry, a quadrupole-linear ion trap instrument was employed. They found 35 differentially regulated proteins suggesting IGLV4-3, lysozyme C, mucin B und lipocalin as potential biomarkers. As lysozyme C was significantly less abundant in CRS patients a deficiency in innate immunity could be suggested. Moreover, lower amounts of phospholipase A2 responsible for the arachidonic acid pathway and less antileukoproteinase A responsible for defense against proteases were quantified in CRS. This suggested impaired anti-inflammatory response and barrier function in CRS patients. Our group was the first to investigate the differences between allergic rhinitis and healthy mucus proteomes. Patients with allergic rhinitis were diagnosed according to skin prick test and radio-allergo-sorbent assays. Non-pollen allergics (house dust mite, animals, etc.) and other related comorbidities (e.g. CRS was excluded) to harmonize the collective. In season and out of season mucus collection was related to symptoms and natural pollen exposure. A special suction device with a mucus trap was used to collect native mucus under endoscopic control. Equal amounts of protein digests were separated by reverse-phase nano-LC and measured online with tandem mass spectrometry on an FT-ICR instrument. Label-free quantitation by spectral counting or precursor intensities (Areas under the curve (AUCs), i.e. mean areas of extracted ion chromatograms of the individual peptides matched to a protein normalized on the total AUC of all proteins in each sample). Identified proteins were annotated by using data from UniProt (www.uniprot.org), the PANTHER classification system (www.pantherdb.org), and DAVID (http://david.abcc.ncifcrf.gov). Enrichment analysis was performed with BINGO/Cytoscape software (www.cytoscape.org). We identified 267 different protein groups with 5 proteins up- and 5 proteins significantly downregulated in allergic rhinitis [65]. In our subsequent study [66] we addressed the analysis of seasonal differences between allergic rhinitis patients and healthy controls. In total, 430 different proteins were detected in both groups, of which 203 (47%) were newly identified.

**Table 1.** Summary of current nasal secretion proteomics studies.

Patients	Sampling	Proteomics technique	Identified proteins	Reference
Chronic rhinosinusitis (CRS) patients (N = 4 and 6) and healthy controls (N = 4 and 6)	Nasal mucus by suction	Isobaric tags (iTRAQ), MRM; QTRAP	35 (7 validated)	Tewfik [63], Badaai [64]
Allergic rhinitis patients (N = 29) and healthy controls (N = 29)	Nasal mucus by suction	Spectral counting; LTQ-FT-ICR	267	Tomazic [65]
Allergic rhinitis patients (N = 10) and healthy controls (N = 12) in and out of season	Nasal mucus by suction	Label free quantitation of precursor ion intensities; LTQ-FT-ICR	430	Tomazic [66]
Healthy controls (N = 10)	NLF	Identification; Q-TOF	110	Casado [6]
Allergic rhinitis patients (N = 6) and healthy controls (N = 5)	NLF	2DE, MALDI-TOF	20	Ghafouri [81]
Nonpolypoid AR (N = 9) and CRS with coexisting asthma (N = 6)	NLF	Identification; LTQ	202 and 163	Benson [82]
Allergic rhinitis patients (N = 40)	NLF	Isobaric tags (TMT); LTQ-Orbitrap	953	Wang [83]
Allergic and nonallergic rhinitis patients (N = 8 and 7) and healthy controls (N = 7)	Nasal fluid by swab	MALDI-TOF profiling	-	Lombardo [85]
Allergic rhinitis patient (N = 1) and healthy controls (N = 8)	Nasal fluid by swab	MALDI-TOF profiling	11	Preiano [84]
CRS patients (N = 10 with and 10 without nasal polyp) and healthy controls (N = 10)	Nasal fluid by filter paper	DDA (label free quantitation of precursor ion intensities) and DIA; Q-Orbitrap	2020	Kim [86]

**Figure 1.**
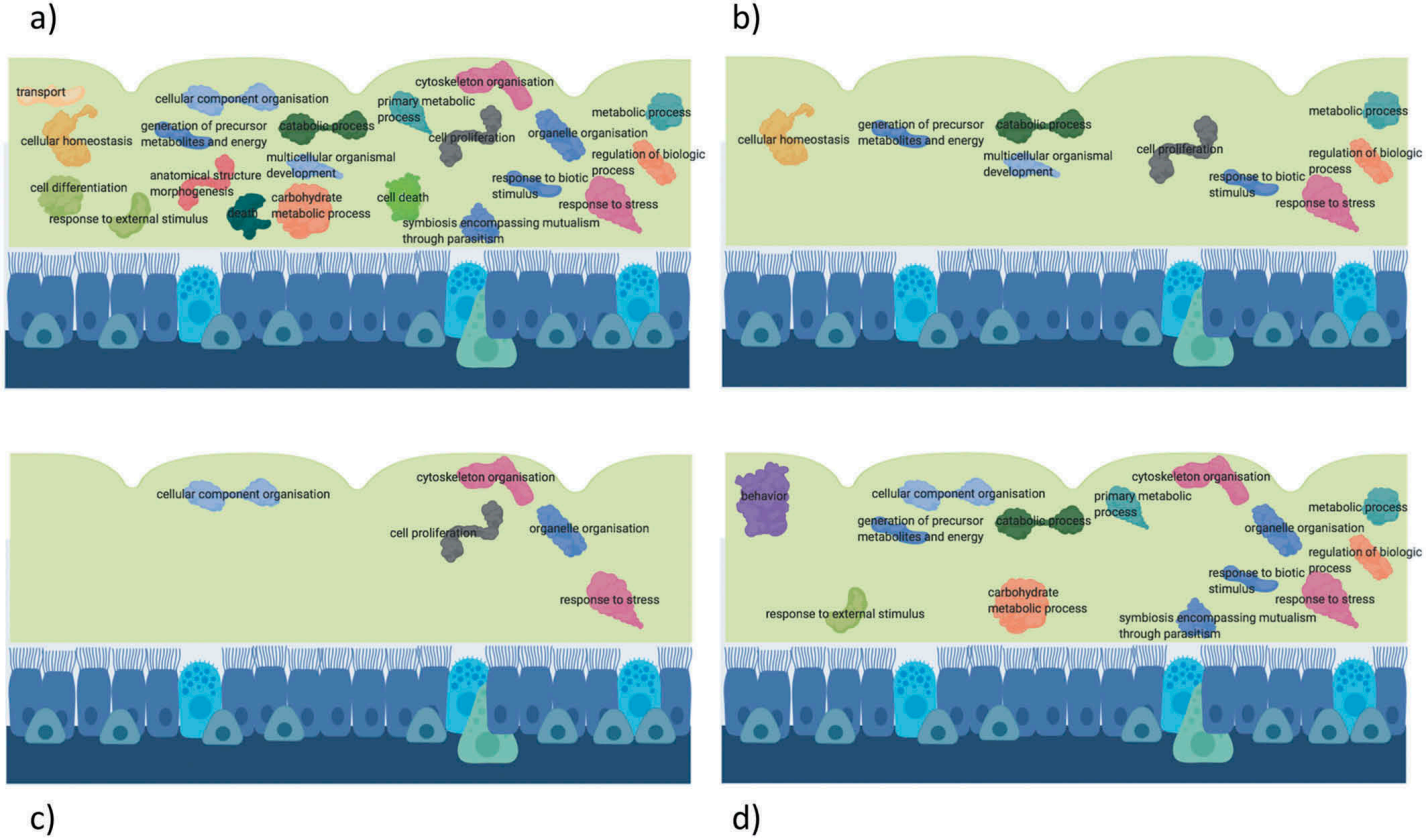
Significantly enriched biological processes compared to total human proteome in healthy controls (HC) in season (a), in allergic patients (AR) in season (b) as well as in HC out of season (c) and in AR out of season (d).

Our studies show that the nasal mucus proteome differed between groups as well as seasons: Allergic rhinitis patients had an activated inflammatory state of the nasal mucus proteome throughout the year peaking outside the pollen season contrary to their symptoms. Healthy controls showed a highly regulated state of the proteome during the season reflecting enhanced defense mechanisms against natural pollen exposure (Figure 1).

Our data suggest that the allergic rhinitis mucus proteome, contrary to the absence of symptoms out of season, reflects a perennial inflammatory response increasing during pollen season despite reduced defense mechanisms of the immune system and reduced integrity of the epithelial barrier. Interestingly, the nasal mucus of allergic rhinitis patients contained significantly fewer distinct proteins exclusively in season compared to healthy controls (68 vs. 124) resembling a higher diversity of abundant proteins in healthy controls as a reaction to pollen exposure. The numerical change of different proteins exclusively present in as compared to out of season was markedly higher in healthy controls (124 vs. 38) compared to allergic rhinitis patients (68 vs. 48), normalized on total protein amount. These data suggest a higher plasticity of the healthy compared to the allergic mucus proteome. When comparing the nasal mucus proteome of allergic rhinitis patients and healthy controls in pollen season 10 proteins were found to be significantly more abundant in allergic rhinitis. Of these, complement C4-B (C4B), alpha-1-acid glycoprotein 2 (ORM2) and phospholipid transfer protein (PLTP) were exclusively present in allergic rhinitis; while alpha-2-macroglobulin (A2M), apolipoprotein A-II (APOA2), vitamin D-binding protein (GC), complement C3 (C3), apolipoprotein A-I (APOA1), BPI fold-containing family B member 2 (BPIFB2), and clusterin (CLU) were also found in healthy controls. Most of these proteins are also present in plasma and may indicate therefore increased plasma exudation [67,68] and disintegration of the epithelial barrier in allergy [69–71]. C3, A2M, APOA1, and APOA2 were also found to be significantly more abundant in nasal mucus in allergic rhinitis patients in our previous studies [65,72]. Apolipoproteins could act as anti-inflammatory agents apart from their involvement in lipid metabolism [73,74] and their high abundance could be regulated by a local mechanism since plasma levels of APOs did not differ between allergic rhinitis patients and healthy controls and did not correlate to nasal mucus levels [72]. However, further studies are needed to investigate the local production of APOs in the epithelium as a possible alternative mechanism to increased plasma exudation. On the other hand, APOs could also act pro-inflammatory through posttranslational modifications and complexation with other molecules in chronic inflammation [75]. PLTP is responsible for phospholipid transfer between lipoproteins and is elevated in plasma during acute inflammation. Interestingly, it binds to apolipoprotein A-I, apolipoprotein E, and clusterin and forms complexes involved in immune response [76]. C3, C4B, ORM2 [77], BPIFB2 [51,78,79] and GC [80] are pro-inflammatory molecules acting over different ways like the complement system or innate immune cells. As ORM2 was newly identified in the nasal mucus in our study, its role in the upper airway tract needs to be elucidated.

Apart from obtaining native nasal mucus other sampling methods of nasal secretions for mass spectrometry-based proteomic analysis have been employed: a common way is to obtain nasal lavage fluid (NLF). Casado et al. identified 110 different proteins in NLF of a healthy collective. NLF was collected twice after provocation with saline, digested with trypsin and subjected to a data-dependent identification workflow employing reverse-phase capLC coupled to tandem mass spectrometry by electrospray ionization on a quadrupole-time of flight (Q-TOF) instrument. The majority of identified proteins were of plasmatic origin and secondly of local glandular origin. Their functions were mainly dedicated to cytoskeleton organization followed by innate and adaptive immune regulation. One interesting protein group identified by the authors was the antimicrobial PLUNC proteins. Since the functional properties in these healthy subjects’ proteins were predominantly related to immune response their comparison to patients could shed light on defective immune reactions and subsequent disease [6]. Ghafouri et al. [81] collected NLF from six patients with seasonal allergic rhinitis and 5 healthy subjects. Proteins were analyzed by two-dimensional gel electrophoresis (2-DE) and matrix-assisted laser desorption/ionization time-of-flight mass spectrometry (MALDI-TOF MS) after tryptic in-gel digest. Twenty proteins could be identified using this approach. PLUNC acting in innate immunity, VEGP and cystatin S, two endogenous proteinase inhibitors, were found to be lower abundant in allergics during season. Contrary alpha-1-antitrypsin was higher in controls in season underlying the importance of endogenous antiproteases as well as innate immunity in allergy as well as CRS.

To identify low-abundance proteins in NLF of patients with allergic rhinitis or chronic sinusitis with asthma Benson et al. [82] used a combination of affinity, anion exchange, and reverse-phase separation techniques to extensively fractionate intact NLF proteins prior to tryptic digestion and nanoLC/MS/MS analysis on a linear ion trap mass spectrometer (LTQ; ThermoFinnigan San Jose, CA).

Wang et al. [83] applied proteomic approaches to NLF analyzing biomarkers for the response to glucocorticoids in 40 patients with allergic rhinitis and stratified them in high and low responders. nanoLC-MS/MS analysis was performed on an LTQ Orbitrap Velos instrument (Thermo Fisher Scientific, Inc., Waltham, MA, USA). MS data analysis was performed using Proteome Discoverer version 1.2 and pathway analysis was done with Ingenuity Pathway Analysis.

Apart from NLF nasal secretions can be obtained for mass spectrometric analysis by nasal cotton swabs or filter papers. Preiano et al. [84] and Lombardo et al. [85] used cotton swabs and a fast procedure based on mesoporous silica particles (MPS) to enrich nasal fluids in its antimicrobial protein component in combination with MALDI-TOF/TOF MS to differentiate between healthy probands and patients with upper respiratory diseases like allergic rhinitis by proposing ‘rhinomic fingerprints.’

Kim et al. [86] applied filter paper in patients with CRS to collect nasal secretions for nanoLC-MS/MS on a quadrupole orbitrap mass spectrometer (Q-Exactive plus; Thermo Fisher Scientific). They found 2,020 proteins in total by data-dependent acquisition (DDA) and data-independent acquisition (DIA) which showed increased IL-7, IL-9, IL-17A, and IL-22 signaling and neutrophil degranulation and activation in CRS with nasal polyps compared to controls in canonical pathway and in gene ontology (GO) enrichment.

## 3. Conclusion

Nasal mucus proteins are significantly altered in allergic rhinitis and involved in barrier function and inflammatory response. Particularly, the nasal mucus proteome shows a distinct expression profile throughout the season suggesting an active defensive response against pollen exposure in healthy controls which is blunted in allergic rhinitis.

## 4. Expert opinion

Mass spectrometry is a valuable tool for the investigation of the nasal mucus proteome and its role in healthy and diseased state. A global mass spectrometry-based analytical approach followed by cluster analysis of protein groups or families and statistical assessment of changes in abundances or regulations provides an important picture of disease processes. In addition to that the clear advantage of untargeted proteomic techniques using mass spectrometry is the potential to allocate protein identity and quantity without defining the targets beforehand as in ELISA, Western blotting, or other antibody-based targeted techniques. From this unbiased data, future targeted approaches, which can be mass spectrometry (e.g. multiple or parallel reaction monitoring or data-independent acquisition (DIA)) or antibody-based, to validate significantly altered proteins, complemented by functional studies will further elucidate how the respective nasal mucus proteins influence barrier function of the epithelium, immune response, and defense against allergens in allergic rhinitis.

Currently, many studies have focussed on distinct biomarkers, especially interleukins and their downstream action on cells and other mediators. By applying proteomic approaches, a global view of which processes differ between healthy controls and allergic patients enables exploration of a variety of potential biomarkers as well as their interactions rather than focusing on a single or few mediators. Moreover, the analysis of nasal mucus as first-line barrier may shed light on later submucosal processes. A potential target for future studies could be the analysis of defensive proteins like antiproteases. Pollen containing allergens contains a significant amount of exogenic protease that could disrupt mucus and epithelial barrier which can lead to subsequent allergen exposure to immune cells and start sensitization. If those proteases were stopped in the mucus already by innate antiproteases this could prevent the development of allergies as in healthy controls.

The most interesting finding of our studies was that healthy controls showed a high plasticity of their mucus proteins augmented in pollen season suggesting that the altered proteome acts protective against natural pollen stress. In contrary, in allergic rhinitis, the nasal mucus proteome did not react as strikingly and rather showed a perennial inflammatory response despite lack of allergic symptoms out of season. The significantly up-regulated proteins in healthy controls in pollen season could thus harbor the potential for new therapeutic strategies. In the future, the substitution of defective proteins, e.g., as nasal sprays may be an additional therapy of choice.

Proteomic changes during allergen immunotherapy correlated with outcome measures could be used to predict response to treatment and harbor the potential of avoiding therapies without effect. Certain proteomic patterns prior to starting the therapy may indicate the later treatment response. An advantage of investigating nasal mucus as a source for biomarkers is its easy obtainment. Moreover, recently nasal epithelium and its barrier function became the focus of research thus mucus is a direct reflector of pathophysiologic processes occurring in the epithelium. The shortcoming of proteomic approaches is the technical effort of mass spectrometric experiments and subsequent analysis of large datasets, as well as the associated costs. Technological advances in the future in combination with mobile technologies and/or artificial intelligence may provide us with simpler measurements of nasal mucus proteins.

Upon functional analysis in the future the identified significantly altered proteins could serve as novel biomarkers for new diagnostic or therapeutic approaches.

## Article Highlights

- Proteomics of nasal mucus harbors potential for new biomarkers.
- Nasal mucus has an important barrier function against allergens.
- Nasal mucus proteins play an important immunomodulatory role in allergy.
- Nasal mucus proteins reflect seasonal allergen challenge.
- Nasal mucus proteome shows a higher plasticity in healthy controls than patients upon allergen challenge.
